# Mitochondrial Disorder in a Child With Brainstem Lesions Mimicking Thiamine Deficiency

**DOI:** 10.7759/cureus.98909

**Published:** 2025-12-10

**Authors:** Mohamad A Asfour, Jennifer Nedimyer Horner, Kanika Gupta, Gleidson Silva, Tushar Chandra, Gian Rossi

**Affiliations:** 1 Radiology, HCA Healthcare/USF Morsani College of Medicine, Trinity, USA; 2 Radiology, University of Central Florida College of Medicine, Orlando, USA; 3 Radiology, Nemours Children's Hospital, Orlando, USA; 4 Neurology, Nemours Children's Health, Lakeland, USA

**Keywords:** brain, genetic diseases, magnetic resonance imaging, mitochondria, mitochondrial disease, mt-nd5, neuroradiology, oxidative phosphorylation, thiamine deficiency

## Abstract

Mitochondrial diseases are among the most common genetic disorders. Known as the “powerhouse” of the cell, mitochondria generate energy via oxidative phosphorylation, a process that involves five enzyme complexes. The MT-ND5 gene, which encodes part of Complex I, is especially prone to mutations and is linked to various mitochondrial disorders. Since mitochondria are concentrated in metabolically active organs such as the brain, heart, liver, muscles, and kidneys, these systems are particularly vulnerable to dysfunction.

In the brain, mitochondrial disease symptoms often arise in regions with high metabolic demand, such as the brainstem. Disruptions in oxidative phosphorylation due to nicotinamide adenine dinucleotide (NADH)-ubiquinone oxidoreductase chain 5 (MT-ND5) mutations can prevent energy production from meeting cellular demands, leading to serious neurological consequences. This report describes the neuroimaging and clinical presentation of a child with an MT-ND5 pathogenic variant, highlighting characteristic MRI findings and the diagnostic challenges posed by overlapping features with other metabolic disorders, such as thiamine deficiency.

## Introduction

Mitochondrial diseases are among the most common inherited metabolic disorders, impacting an estimated one in 4000 people in the United States and one in 5000 people worldwide [1,2]. Regarded as the “powerhouse” of the cell, the mitochondria are responsible for generating cellular energy through a process called oxidative phosphorylation. This process of converting glucose into the cellular energy that is necessary for aerobic metabolism terminates in a process involving a series of five enzyme complexes. Complex I, known also as the nicotinamide adenine dinucleotide (NADH)-ubiquinone oxidoreductase, is the largest of these complexes and interacts functionally with Complex III and IV. For this reason, genetic mutations resulting in a deficiency of this complex are an important cause of human mortality and morbidity [3].

Although many of these genes have not been well-studied, the MT-ND5 gene, involved in encoding complex I, has been found to be especially susceptible to mutations and has been linked to multiple mitochondrial diseases with varying phenotypic presentations [3]. Organs that are highly metabolically active exhibit a higher concentration of mitochondria; thus, organs such as the heart, brain, liver, muscles, and kidneys often display the greatest susceptibility to mitochondrial deficiency.

For the brain specifically, the neurological manifestations of mitochondrial diseases are often reflective of the involvement of regions that exhibit high metabolic demand, such as the brainstem. Mutations that disrupt the ability of oxidative phosphorylation to meet these metabolic demands, such as those observed in the MT-ND5 gene, are important causes of morbidity in childhood mitochondrial encephalomyopathies [4]. Given the broad phenotypic variability of MT-ND5-related disease and its overlap with other metabolic and neurodegenerative disorders, early diagnosis can be challenging. Although neuroimaging may offer important diagnostic clues, genetic confirmation is frequently required to establish the underlying etiology.

These diagnostic challenges are exemplified in the present case, in which a young child exhibited overlapping radiographic and clinical features that initially suggested thiamine deficiency but were ultimately attributable to an MT-ND5 pathogenic variant. The following case highlights the complexity of interpreting neuroimaging findings in mitochondrial disease and underscores the importance of comprehensive genetic evaluation in pediatric patients with unexplained neurological symptoms.

## Case presentation

A two-year-old boy, born at 35 weeks without complications, initially presented to neurology for concerns of developmental delay and “shivering episodes.” At the time, the patient was still not walking, and speech was significantly delayed. There is a positive family history of speech delays as well as tremors in the patient’s father, paternal grandfather, and uncles. Physical exam revealed hypotonia, long palpebral fissures, saccadic intrusions, restricted ocular abduction, and marked ataxia with intention tremor, titubation, and a wide-based gait. He started physical therapy and received genetic testing.

**Figure 1:**
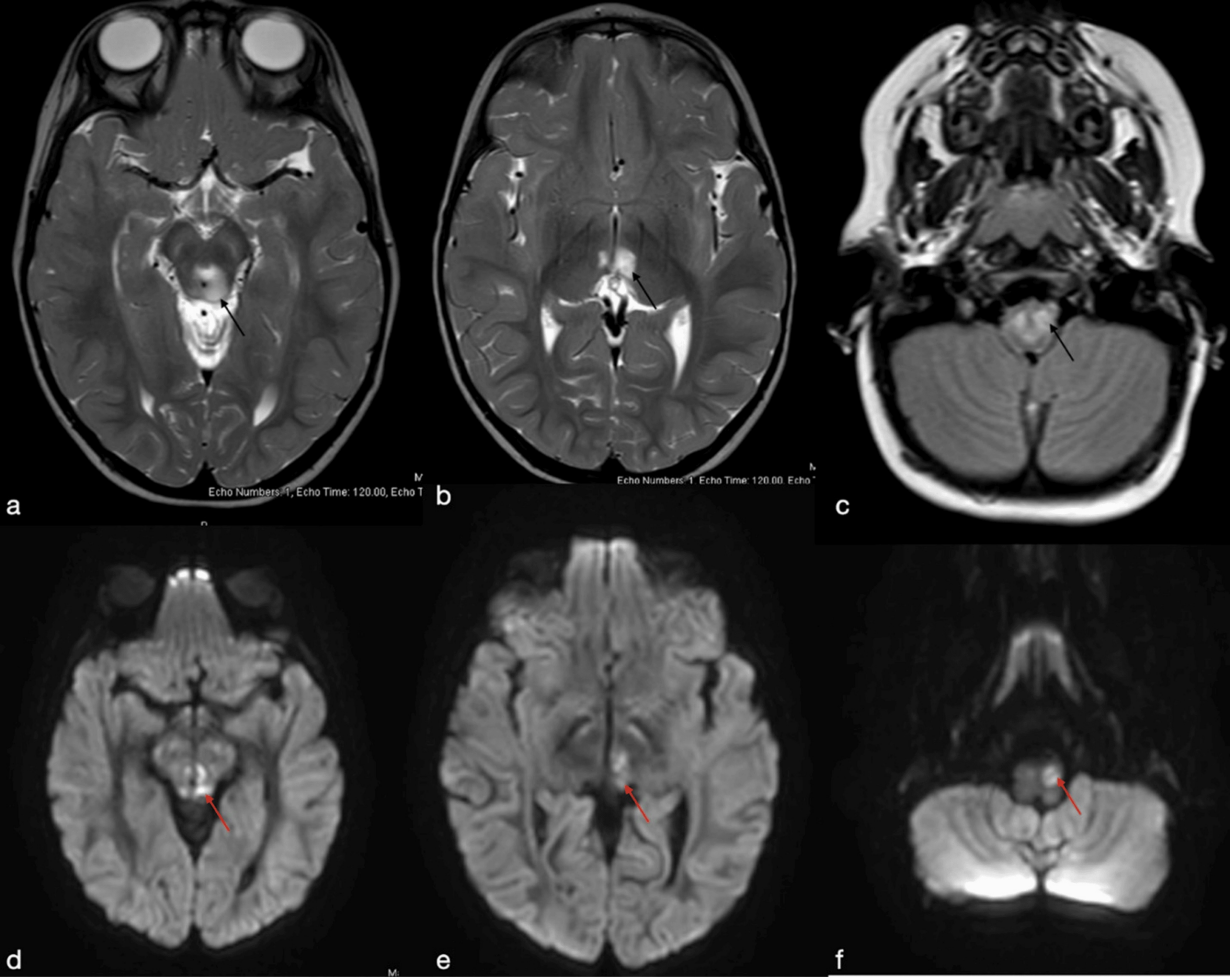
(a and b) T2-weighted axial images showing increased periaqueductal and medial thalami signal respectively (black arrows). (c) FLAIR axial imaging shows increased signal in the upper cervical spine (black arrow). (d-f) Diffusion-weighted imaging shows restricted diffusion in the periaqueductal, medial thalami, and inferior olivary nuclei respectively (red arrows) FLAIR: fluid-attenuated inversion recovery

Over the next few months, the patient began experiencing developmental regression, seizures, and visual changes. Upon presentation to the hospital, the patient underwent evaluations including EEG, MRI, and labs. EEG after one hour did not reveal any epileptiform discharges. Brain MRI revealed extensive abnormal signals involving the upper cervical spine, dorsal aspect of the brainstem in the periaqueductal locations, tectum, bilateral cerebral peduncles, and medial aspect of both thalami (Figure 1). Thiamine deficiency was considered, so the patient was started on replenishment therapy while awaiting further genetic tests. He was then evaluated by ophthalmology and found to have bilateral optic nerve atrophy.

The lab results, a few weeks later, revealed a normal thiamine of 168 mol/L, but his mitochondrial gene panel resulted in a positive MT-ND5 pathogenic variant m.13513 G>A, confirming diagnosis of a mitochondrial disorder for the 62% heteroplasmy. These results were obtained via a combined mito genome plus mito nuclear gene panel/sequencing and deletion analysis of the mitochondrial genome and sequencing and deletion/duplication analysis of nuclear genes. While on thiamine replacement therapy, the mother reported the patient's visual symptoms were improving. The patient was started on a mitochondrial cocktail including B vitamins (B1, B2, B3, B4, B5, B6, and B12), Coenzyme Q10, L-carnitine, and alpha-lipoic acid (ALA). Three months later, the patient's examination was modestly improved with regard to muscle tone and eye gaze ability, but he remains profoundly ataxic.

## Discussion

This case highlights the diagnostic complexity and phenotypic variability of mitochondrial disorders, particularly involving MT-ND5 mutations. Initial presentation, including the radiographic findings of dorsomedial thalami and periaqueductal T2/FLAIR signal, strongly mimicked thiamine deficiency. Meanwhile, lab results of thiamine levels demonstrated normal values and genetic testing confirmed the MT-ND5 mutation. Interestingly, the patient demonstrated some clinical improvement after initiating thiamine therapy and later mitochondrial cocktail therapy, echoing findings from studies suggesting symptomatic benefit even in confirmed mitochondrial disease [5]. This overlap of findings stresses the importance of broadening the differential diagnosis when evaluating pediatric patients with unexplained neurologic symptoms.

The MT-ND5 gene provides instructions for making part of Complex I, a crucial enzyme in the mitochondrial respiratory chain that generates energy for the body. Mutations in the MT-ND5 gene disrupt the function of Complex I, leading to a deficiency in its energy-producing ability. 

These genetic changes are maternally inherited because they are located in the mtDNA, which is passed down from the mother. MT-ND5 mutations can cause a broad range of mitochondrial encephalopathies, such as mitochondrial encephalomyopathy, lactic acidosis, and stroke-like episodes (MELAS), Leigh syndrome, Leber hereditary auditory neuropathy (LHON), or other nonspecific neurodegenerative syndromes [3]. The metabolically demanding regions, such as the brainstem and thalami, seen on MRI such as in this case, suggested mitochondrial dysfunction.

When compared with other published MT-ND5 cases, this child’s presentation is consistent with the recognized phenotypic diversity and incomplete genotype-phenotype correlations associated with this gene. Ng et al. reported that even low mutant loads in MT-ND5 can be associated with highly variable neurological manifestations, ranging from Leigh-like syndromes to MELAS-like or nonspecific neurodegenerative phenotypes [3]. Similarly, Sonam et al. described two children with MT-ND5 mutations who exhibited a spectrum of clinical features, including developmental delay, ataxia, and optic involvement, further illustrating that the same gene can underlie distinct clinical syndromes [6]. Our patient’s combination of developmental regression, ataxia, optic atrophy, and brainstem-predominant lesions fits within this broad continuum but does not align neatly with a single classic mitochondrial syndrome, highlighting the limitations of traditional phenotype-based classifications.

Neuroimaging complemented by genetic testing is essential in evaluating mitochondrial disease. The highly metabolic brain structures, such as those affected in this case, are extremely susceptible to damage in such diseases. Optic nerve atrophy, developmental regression, and delayed milestones in this case are all consistent with prior cases of MT-ND5 presentations [6]. Ongoing multidisciplinary care and genetic counseling are essential for disease management and prognosis.

## Conclusions

This case highlights the diagnostic challenges posed by MT-ND5 mutations, which can mimic other metabolic disorders such as thiamine deficiency. As seen in previously published MT-ND5 cases, wide genotype-phenotype variability and diverse MRI patterns limit the reliability of imaging alone. Comprehensive genetic testing is therefore essential when evaluating children with unexplained neurologic regression or symmetric deep gray matter lesions. Although no curative therapy exists, supportive treatments, such as mitochondrial cocktails and vitamin supplementation, may offer symptomatic benefit. Early recognition, multidisciplinary care, and genetic counseling remain central to optimizing outcomes in MT-ND5-associated mitochondrial disease.
